# Stereotactic body radiotherapy with CyberKnife for liver-confined hepatocellular carcinoma: systematic review and meta-analysis

**DOI:** 10.1093/jrr/rraf040

**Published:** 2025-07-24

**Authors:** Takanori Abe, Mototaro Iwanaga, Mitsunobu Igari, Ryuta Hirai, Yu Kumazaki, Shin-ei Noda, Shingo Kato

**Affiliations:** Department of Radiation Oncology, International Medical Center, Saitama Medical University, 1397-1, Yamane, Hidaka, Saitama 350-1298, Japan; Department of Radiation Oncology, Kanto Neurological Surgical Hospital, 1120, Dai, Kumagaya, Saitama 360-0804, Japan; Department of Radiation Oncology, International Medical Center, Saitama Medical University, 1397-1, Yamane, Hidaka, Saitama 350-1298, Japan; Department of Radiation Oncology, International Medical Center, Saitama Medical University, 1397-1, Yamane, Hidaka, Saitama 350-1298, Japan; Department of Radiation Oncology, International Medical Center, Saitama Medical University, 1397-1, Yamane, Hidaka, Saitama 350-1298, Japan; Department of Radiation Oncology, International Medical Center, Saitama Medical University, 1397-1, Yamane, Hidaka, Saitama 350-1298, Japan; Department of Radiation Oncology, International Medical Center, Saitama Medical University, 1397-1, Yamane, Hidaka, Saitama 350-1298, Japan

**Keywords:** Cyberknife, stereotactic body radiotherapy, hepatocellular carcinoma, systematic review, meta-analysis

## Abstract

The purpose of this systematic review and meta-analysis was to evaluate the efficacy and safety of stereotactic body radiotherapy using CyberKnife (CK) for liver-confined hepatocellular carcinoma (HCC). A systematic review was performed on studies published between 2000 and 2024 that reported treatment outcomes including overall survival (OS), local control (LC), and complications. A literature search was performed using MEDLINE/PubMed with the following terms: (‘Carcinoma, Hepatocellular’[MeSH Terms] OR ‘hepatocellular carcinoma’ OR ‘HCC’) AND (‘CyberKnife’ OR ‘robotic radiosurgery’). Additional searches were conducted on Scopus and the Cochrane library using following terms: ‘Hepatocellular carcinoma’ AND ‘CyberKnife.’ A meta-analysis was performed to assess OS and LC using weighted random effects models. Five retrospective studies and one prospective study were included in the meta-analysis, comprising a total of 697 patients with a median follow-up duration was 31 months (range: 15–48 months). The pooled 3-year LC and OS rates were 82.5% (95% confidence interval [CI], 75.0%–90.0%) and 58.7% (95% CI, 47.2%–70.1%), respectively, which is comparable to previous reported outcomes for non-device-limited SBRT and similar to that of surgery and local ablative therapies. The incidence of radiation-induced liver disease was 4.3%–15.3%. Stereotactic body radiotherapy using CK appears to be an effective and well-tolerated treatment for liver-confined HCC. However, further prospective studies with standardized methodologies are warranted to establish solid evidence of its clinical utility.

## INTRODUCTION

Hepatocellular carcinoma (HCC) was the sixth most frequently diagnosed cancer and the third leading cause of cancer-related deaths worldwide in 2022 [1]. Standard treatments for liver-confined HCC include surgery or local ablative therapies such as radiofrequency ablation (RFA) or microwave ablation (MWA). Stereotactic body radiotherapy (SBRT) is increasingly recognized as a viable treatment option for HCC [2]. SBRT is a minimally invasive treatment that can be performed on inoperable patients with comorbidities and on patients whose tumors are in anatomically challenging locations for local ablative therapy. A meta-analysis by Bae *et al*. reported 5-year local control (LC) and overall survival (OS) rates of 89% and 51%, respectively, for HCC < 3 cm treated with SBRT, outcomes comparable to those of surgical resection and local ablative therapy [3]. SBRT delivers highly precise radiation beams from multiple directions, concentrating the radiation dose on the tumor while minimizing radiation exposure to surrounding normal tissue. The conventional SBRT system is a gantry-based linear accelerator (linac), which uses isocentric non-coplanar beams or volumetric modulated arc therapy technique to concentrate the dose to the tumor. Conversely, CyberKnife (CK) is a specialized SBRT system that features a linear accelerator attached to a robotic arm, allowing irradiation of non-isocentric beams from ultra-multi directions [4]. In this setting, maximum dose tends to be high compared with conventional linac with lowering dose to surrounding normal tissue. CK can also perform respiratory motion tracking irradiation using metallic fiducial markers, reducing radiation field expansion caused by respiratory motion [5]. These features may be particularly advantageous for the treatment of HCC with SBRT [6, 7]. However, most reports on SBRT for HCC have focused on linac-based SBRT, while reports on SBRT using CK (CK-SBRT) are primarily retrospective with limited patient numbers. To our knowledge, no systematic reviews or meta-analyses have specifically examined treatment outcomes CK-SBRT for HCC. Therefore, in this study, we conducted a systematic review and meta-analysis to examine patient characteristics, radiation dose, survival rate, local control rate, and incidence of adverse events in CK-SBRT for HCC.

## MATERIALS AND METHODS

### Overview of the review process

This systematic review was conducted according to Preferred Reporting Items for Systematic Review and Meta-Analyses guidelines [8]. The protocol was registered at https://www.crd.york.ac.uk/PROSPERO/ (Review registry: CRD42025641765). The purpose of the systematic review was outlined using the Population, Intervention, Comparison, Outcomes, and Study framework, as shown in Table 1.

**Table 1:** Population, Intervention, Comparison, Outcome, and Study (PICOS) criteria

P (Patient/Population)	Patients with liver-confined hepatocellular carcinoma
I (Intervention)	Stereotactic body radiotherapy using CyberKnife
C (Comparison)	None (including single-arm studies) or other treatments
O (Outcome)	Overall survival rate, local control rate, incidence of adverse events
S (Study design)	Randomized controlled trials, observational studies, and cohort studies

### Literature search

A literature search was performed on MEDLINE/PubMed using the following terms: (‘Carcinoma, Hepatocellular’[MeSH Terms] OR ‘hepatocellular carcinoma’ OR ‘HCC’) AND (‘CyberKnife’ OR ‘robotic radiosurgery’). Additional searches were conducted on Scopus and the Cochrane library using the terms ‘Hepatocellular carcinoma’ AND ‘CyberKnife.’ Studies published in English from 2000 to 2024 were included. Additionally, the manufacturer of Cyber Knife, Accuray Incorporated (Sunnyvale, CA, USA) was contacted to inquire about relevant literature on CK-SBRT for HCC.

### Screening

After the initial search, duplicate studies were removed. Two independent reviewers (T.A. and M.I.) then screened the remaining studies by title and abstract to exclude non-relevant studies, including case reports, radiobiological studies, studies on metastatic liver tumors, and studies dealing with medical physics. Next, full-text analysis was performed based on pre-specified inclusion and exclusion criteria. Prospective and retrospective studies of CK-SBRT for liver-confined HCC were considered eligible for inclusion. For this study, SBRT was defined as radiotherapy delivered in 1–10 fractions with a minimum dose of 5 Gy per fraction. The following exclusion criteria were applied: SBRT used as bridging, neoadjuvant, or adjuvant therapy, SBRT combined with other treatments within 1 month, studies lacking survival or local control data, studies focused on HCC with portal vein tumor thrombosis, studies with fewer than nine patients, and SBRT administered as re-irradiation. If multiple studies appeared to report on the same patient cohort, the study with the largest sample size was included in the review.

### Risk of bias assessment

Two reviewers independently assessed study quality using the revised Newcastle-Ottawa scale (NOS) for cohort studies [9]. Studies with a score of 7 to 9 were considered high quality, while those scoring 4 to 6 were considered medium quality. Publication bias was evaluated using funnel plots and the Egger’s regression test. A symmetrical funnel plot or a *P* value >0.05 in Egger’s test indicated no significant publication bias.

### Data extraction and synthesis

The following data were extracted and recorded: (i) patient and tumor characteristics, (ii) treatment characteristics, (iii) OS and LC rate, and (iv) hepatic toxicity rates. Hepatic toxicity was classified according to the Common Terminology Criteria for Adverse Events. Radiation-induced liver disease (RILD) was categorized as either classic RILD or non-classic RILD [10]. Survival and local control results from comparative and noncomparative studies were pooled using the inverse variance method. The I^2^ test was used to measure heterogeneity [11]. If significant heterogeneity was present, a random-effects meta-analysis for the primary analyses was applied.

## RESULTS

### Search and screening results

**Fig. 1:**
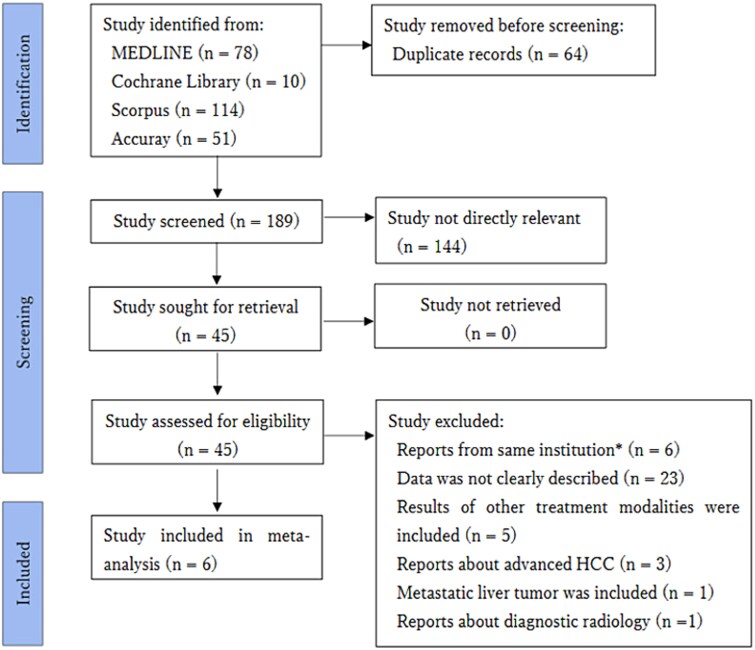
PRISMA (Preferred Reporting Items for Systematic Reviews and Meta-Analyses) flowchart of study selection process was shown. An initial search of four databases identified 343 studies. After screening by two reviewers (TA and MI), a total of six studies were considered to be suitable for meta-analysis.

An initial search of four databases identified 253 studies. After removing 64 duplicate studies, 189 studies underwent title and abstract screening. At this stage, 144 non-relevant studies were excluded. The remaining 45 studies underwent full-text review, resulting in the exclusion of 39 studies for the following reasons: reports from same institution (*n* = 6), data were not described (*n* = 23), use of other treatment modalities (*n* = 5), reports about HCC with portal vein tumor thrombosis (*n* = 3), metastatic liver tumors (*n* = 1), and reports about diagnostic radiology (*n* = 1). These results are shown in Fig. 1. Ultimately, a total of six studies were identified.

### Characteristics of identified studies

Five retrospective studies and one prospective study were included in the meta-analysis. Most of the studies were conducted in Asia and one was conducted in France. A total of 697 patients were analyzed, with a median age of 59 years old (range: 22–91). The proportion of tumors with viral etiology ranged from 25% to 100%, with higher rates in studies from Asia. The median tumor size was 2.8 cm (range: 0.5–9.5 cm), and the median proportion of Child-Pugh class A was 86% (range: 46%–94%). The median SBRT dose and fraction was 45 Gy in three fractions. Study details and treatment outcomes are summarized in Tables 2 and 3 [12–17].

**Table 2:** Patient characteristics

**First author**	**Year**	**Location**	**Time of study**	**Study type**	**No.**	**Male patients (%)**	**Median age (range)**	**Tx naïve (%)**	**Viral etiology**	**Proportion of CP class A**	**ALBI grade A/B/C**
Durand-Labrunie *et al.* [12]	2024	France	2009–2014	P2	43	81	72 (43–91)	100	25	86	13/25/3
Seo *et al.* [13]	2010	Korea	2003–2008	R	38	63	61 (37–81)	NR	NR	89	NR
Shen *et al.* [14]	2019	Taiwan	2008–2017	R	46	76	64 (37–86)	26.1	85	86	24/20/3
Sun *et al.* [15]	2024	China	2011–2019	R	520	77	55 (26–88)	100	95	94	155/349/16
Yuan *et al.* [16]	2013	China	2006–2011	R	22	82	57 (43–80)	NR	NR	46	NR
Zhang *et al.* [17]	2018	China	2011–2012	R	28	75	49 (22–65)	36	100	86	NR

*Abbreviations:* Tx = treatment; CP=Child-Pugh; ALBI = albumin-bilirubin; P2 = prospective phase 2 study; R = retrospective study; NR = not reported.

**Table 3:** Tumor characteristics and treatment outcomes

**First author**	**Median size (range) (cm)**	**BCLC stage A/B/C/D**	**Median SBRT dose (Gy)**	**Median no. of fx**	**Median BED**_**10**_ **Gy**	**Median follow-up (mo)**	**LC rate at 1/3/5 y (%)**			**OS rate at 1/3/5 y (%)**		
Durand-Labrunie *et al.* [12]	2.8 (1.0–6.0)	NR	45	3	112.5	48	98	94	NR	87	53	NR
Seo *et al.* [13]	NR	NR	51	3	137.7	15	78.5	66.4	NR	68.4	42.1	NR
Shen *et al.* [14]	5.3 (3.0–7.9)	9/13/22/2	45	5	85.5	26.6	91.3	73.3	NR	73.4	47.4	NR
Sun *et al.* [15]	2.3 (0.5–5.0)	NR	NR	NR	NR	41	100	87.8	87.8	100	69.8	69.8
Yuan *et al.* [16]	4.3 (1.6–9.5)	NR	45	5	85.5	23.5	92.9	67.7	NR	72.7	57.1	NR
Zhang *et al.* [17]	2.1 (1.1–3.0)	NR	NR	NR	NR	36	96.4	89.3	NR	92.9	78.6	NR

*Abbreviations:* BCLC=Barcelona Clinic Liver Cancer; SBRT = Stereotactic body radiation therapy; fx = fractions; BED_10_; biologically effective dose which is calculated using α/β ratio of 10; LC = Local control; OS = overall survival; NR = not reported.

### Bias assessment

The median NOS score was 7 (range, 6–8), indicating medium-to-high study quality. Egger’s test *P*-values for 3-year LC and OS were 0.1996 and 0.1407, respectively. Funnel plot results are provided in Appendix 1.

### Treatment outcomes

**Fig. 2:**
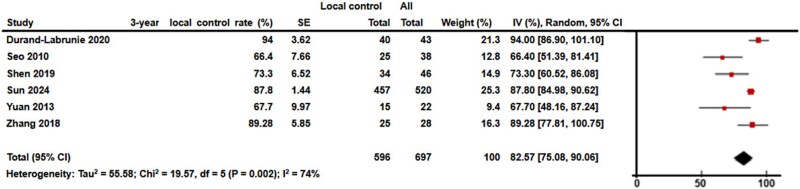
Forest plot of 3-year local control (LC) was shown. The pooled 3-year LC was 82.5% (95% CI, 75.0%–90.0%).

**Fig. 3:**
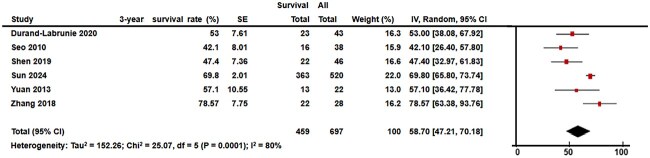
Forest plot of 3-year overall survival (OS) is shown. The pooled 3-year OS was 58.7% (95% CI, 47.2%–70.1%).

The meta-analysis included 697 patients with a median follow-up time of 31 months (range: 15–48). The median 3-year LC and 3-year OS rates were 80.5% (range: 66.4%–94.0%) and 55.0% (range, 42.1%–78.5%), respectively. The I^2^ test results for LC and OS were 74% and 84%, respectively, prompting the use of a random-effects model for data synthesis. The pooled 3-year LC and OS rates were 82.5% (95% confidence interval [CI], 75.0%–90.0%) and 58.7% (95% CI, 47.2%–70.1%), respectively (Figs 2 and 3).

### Toxicity

One study reported incidences of classical and non-classical RILD of 4.3% and 15.3%, respectively [14]. Another study reported an overall incidence of RILD of 7.9% without distinguishing between classical and non-classical RILD [15]. Increases in Child-Pugh score of more than two points within three months post-SBRT was observed in 10% to 15% of patients [12, 14]. Toxicity data are summarized in Table 4.

**Table 4:** Toxicity

	**Acute toxicity**			**Late toxicity**
**First author**	**CTCAE grade ≥ 3 (%)**	**Classical RILD (%)**	**Non-classical RILD (%)**	**CTCAE grade ≥ 3 (%)**
Durand-Labrunie e*t al.* [12]	21	NR	NR	NR
Seo *et al.* [13]	0	0	NR	0
Shen *et al.* [14]	NR	4.3	15.3	NR
Sun *et al.* [15]	0	7.9^a^	NR	NR
Yuan *et al.* [16]	NR	NR	NR	NR
Zhang *et al.* [17]	3.6	NR	NR	NR

*Abbreviations:* CTCAE = Common Terminology Criteria for Adverse Events; RILD = Radiation-induced liver disease; NR = not reported.

^a^Sun *et al.* reported that incidence of RILD was 7.9% without distinguishing classical and non-classical RILD.

## DISCUSSION

To our knowledge, this study represents the first systematic review and meta-analysis of CK-SBRT for liver-confined HCC. A total of six studies were identified, with 697 cases included in the meta-analysis. The median tumor diameter was 2.8 cm, and most frequent SBRT dose was 45 Gy in three fractions. The pooled 3-year LC rate was 82.5%, the pooled 3-year OS rate was 58.7%, and the incidence of RILD ranged from 4.3% to 15.3% [14, 15], which is comparable to results reported in a systematic review and meta-analysis of non-device-limited SBRT [3], as well as to surgery and local ablative therapy [15, 18].

The pooled 3-year OS rate of 58.7% in this study closely aligns with the 57% reported in Bae *et al.*’s meta-analysis of non-device-limited SBRT [3]. In that study, tumor size was a significant factor influencing OS [3]. The median tumor size in our meta-analysis was 2.8 cm, identical to that reported by Bae *et al.*, which may explain the similar OS rates between studies. In reports of surgical outcomes for small HCC, a systematic review showed a 3-year OS rate of 61.9% [18] and a nation-wide cohort study in Japan reported 3-year OS rate of 72–79% [19]. The results of our report are almost the same as those of the systematic review [18] and slightly lower than those of the cohort study [19], but considering that SBRT is often performed in inoperable patients, we believe that our results are comparable to those of surgical outcome. A meta-analysis of RFA, and MWA for small HCC reported 3-year OS rates of 72.8% and 69.1%, respectively [18]. The OS rate for RFA and MWA were slightly better. In clinical practice, HCC treatment decisions are determined through established guidelines and treatment algorithms [20, 21]. Given that the role of SBRT remains unclear in current guidelines, SBRT is often reserved for cases where local ablative therapy is not feasible [20]. Despite the potentially more complex patient population included in our study, the 3-year OS rate observed in this meta-analysis was comparable to those of RFA and MWA, with only slight differences. This suggests that CK-SBRT achieves treatment outcomes similar to other established modalities.

The pooled 3-year LC rate was 82.5%, again consistent with the 84% reported in Bae *et al.* meta-analysis of non-device-limited SBRT [3]. According to a report by Cho *et al*., the local control rate of surgery or RFA for small HCC is 70–90% [22]. In addition, a retrospective study by Livraghi *et al*. on RFA for HCC smaller than 2 cm reported a local control rate of 97.2% over a 31-month of median follow-up period [23]. However, in a report on the treatment outcomes of RFA including HCC up to 5 cm by Mukund *et al.*, the 3-year local control rate was decreased to 80% [24]. Although there was no data by tumor size in this study, tumors up to 10 cm in size were included, and the local control rate is considered to be comparable to that of surgery or RFA. Previous studies have indicated that LC rates in SBRT are associated with the biological effective dose (BED) [25–28]. Reports analyzing BED and LC have demonstrated a 3-year LC rate ranging from 66% to 96% when BED exceeds 100 Gy [25–27]. In the present study, median BED was 106 Gy and the corresponding 3-year LC rate of 82.5% aligns with these findings [25–27]. However, some reports suggest that LC could be further improved with higher doses, particularly BED >150 Gy [26]. None of the studies in our meta-analysis used high doses of BED >150 Gy, so the safety and efficacy of high-dose CK-SBRT remains a topic for future study.

Regarding comparison of toxicity between CK-SBRT and RFA, increases in Child-Pugh score of more than two points within 6 months after CK-SBRT were observed in 12% of patients in a prospective study [12], while that was observed in 15% in a multi-institutional retrospective study of RFA for HCC by Wang *et al.* [29]. It was reported that grade 3 or higher treatment related complication was observed in 2% of patients in CK-SBRT [12] while major complication was observed in 4% of patients in RFA [30]. We believe that these data indicate that SBRT and RFA are similarly less invasive treatment for HCC. Comparison of toxicity between CK-SBRT and surgery is a difficult issue because treatment procedure and patient backgrounds are quite different. In general, SBRT can be performed on an outpatient basis, and as mentioned above, adverse events are within the tolerable range, making it suitable for inoperable patients [2, 20]. Regarding RILD, one study in our analysis reported an incidence of 4.3% for classical RILD and 15.3% for non-classical RILD [14]. Bae *et al.*’s meta-analysis reported classical RILD and non-classical RILD incidences ranging from 0% to 16% and 0% to 25%, respectively [3], indicating that the rates observed with CK-SBRT in our study fall within the expected range. Theoretically, the CK, with its fiducial marker tracking system, should enable precise respiratory motion tracking, thereby minimizing radiation field expansion and improving normal liver sparing as compared with classic SBRT [5–7]. The lack of a demonstrable reduction in adverse events in this meta-analysis may be attributed to inconsistencies in the use of respiratory tracking among the included studies, as most did not specify whether this technique was used. Because marker insertion requires specialized skills and experience, future prospective clinical trials using standardized protocols for marker placement, target definition, and tracking methodology are necessary to fully evaluate the potential benefits of CK’s dose distribution in reducing treatment-related toxicity.

Several limitations of the present study should be noted. First, missing data regarding key patient characteristics, such as Albumin-Bilirubin grade and Barcelona Clinic Liver Cancer stage, introduce some uncertainty regarding the composition of the pooled patient population. Second, detailed information on SBRT methods were lacking in some studies, making it difficult to assess the full capabilities of CK. Third, the majority of included studies were retrospective, increasing the risk of confounding factors and selection bias in the meta-analysis.

In conclusion, this meta-analysis suggests that CK-SBRT provides comparable treatment outcomes to classic SBRT, surgery, and local ablative therapies with an acceptable toxicity profile. Further prospective studies employing standardized treatment methodologies are warranted to establish stronger evidence for CK-SBRT in the treatment of liver-confined HCC.
